# Realizing the AF4-UV-SAXS on-line coupling on protein and antibodies using high flux synchrotron radiation at the CoSAXS beamline, MAX IV

**DOI:** 10.1007/s00216-023-04900-7

**Published:** 2023-08-12

**Authors:** Hans Bolinsson, Christopher Söderberg, Fátima Herranz-Trillo, Marie Wahlgren, Lars Nilsson

**Affiliations:** 1 Department of Food Technology, Engineering and Nutrition, Lund University, Lund, Sweden; 2 RISE Research Institutes of Sweden, Division Bioeconomy and Health, Chemical Process and Pharmaceutical Development, Lund, Sweden; 3 CoSAXS Beamline, MAX IV Laboratory, Lund, Sweden

**Keywords:** AF4-UV-SAXS on-line coupling, AF4, SAXS, Protein, Monoclonal antibody

## Abstract

In this paper, we demonstrate the coupling of synchrotron small angle X-ray scattering (SAXS) to asymmetrical flow-field flow fractionation (AF4) for protein characterization. To the best of our knowledge, this is the first time AF4 is successfully coupled to a synchrotron for on-line measurements on proteins. This coupling has potentially high impact, as it opens the possibility to characterize individual constituents of sensitive and/or complex samples, not suited for separation using other techniques, and for low electron density samples where high X-ray flux is required, e.g., biomolecules and biologics. AF4 fractionates complex samples in native or close to native environment, with low shear forces and system surface area. Many orders of magnitude in size can be fractionated in one measurement, without having to reconfigure the experimental setup. We report AF4 fractionations with correlated UV and statistically adequate SAXS data of bovine serum albumin and a monoclonal antibody and evaluate SAXS data recorded for the two protein systems.

**Graphical Abstract:**

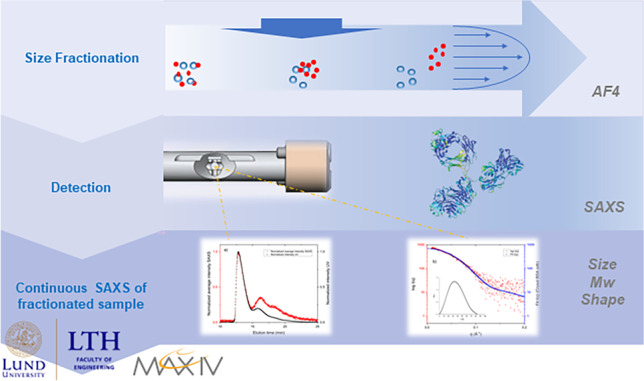

## Introduction

BioSAXS is a common abbreviation for small angle X-ray scattering (SAXS) of biological materials and is used to study structure and average shape and size of biological molecules in solution. This makes it a demanded technique for scientists within the life sciences. In this study, we utilized the BioCUBE at the CoSAXS [1] beamline of MAX IV Laboratory, Lund University, to perform a proof of concept of the on-line coupling between asymmetrical flow-field flow fractionation (AF4) and SAXS. The use of AF4, a technique used for fractionation of colloidal and biological samples in solution, places this conceptually in the application range of BioSAXS.

Since scattering from a sample in solution delivers the sum-weighted contribution of each species to the structural parameters extracted, there is a growing need for separation of sample constituents prior to SAXS data collection, especially for the study of more complex multicomponent samples. To get specific and high-quality data from SAXS in batch mode, it is required to prepare monodisperse samples without contaminants [2]. To perform a study of the properties of a specific analyte in a multicomponent sample becomes difficult or even impossible in batch mode SAXS. Tools exist for the fitting of scattering data from polydisperse systems [3, 4], but the recommendation is to use these tools when fractionation for any reason is not possible, e.g., for cases when the sample would degrade or deteriorate by the separation techniques, or when access to a suitable separation technique is not possible. To achieve monodisperse data from SAXS measurements of multicomponent samples, it is recommended to separate prior to analysis. For this reason, the use of size exclusion chromatography (SEC) in conjunction with SAXS has been elaborated on for some time and was first reported in 2004 [5], where the experimental and data handling circumstances were thoroughly described. Several papers [6–9] on this topic have been published and the technique has been continuously developed and optimized since then. Automatic data handling software has also been made accessible, and individual or collected frames from the chromatograms can be analyzed according to state-of-the-art procedures [3, 10]. The use of chromatographic techniques on-line with SAXS enables background data capture during the measurement since there are windows when only carrier liquid/mobile phase is eluting. The general SAXS data collection procedure is rationalized.

AF4 is a non-column size-based analytical separation technique with a very wide separation range (approx. 2–1000 nm in Brownian mode separation). The coupling of SAXS to AF4 enables characterization of length scales from above 1000 nm down to 1 nm, covering the whole size-range of fractionated analytes from AF4. The fractionation in AF4 is performed in a channel void of stationary phase and retention depends on an analyte’s diffusion coefficient (i.e., hydrodynamic size) through its average height position in the channel, where on the one hand it is forced towards a semi-permeable accumulation wall and on the other hand diffusion brings it away from the accumulation wall. In a parabolic laminar flow profile, with the highest longitudinal flow velocity in the center of the channel, the analyte with the highest average position will elute first. The detailed theory of AF4 can be found elsewhere [11–13]. In SEC, the fractionation is based on the analyte’s pathway through a close packed column with pores; the smaller the analyte, the longer the pathway, which determines retention. As the AF4 channel is void of stationary phase, the shear forces and the interacting surface area of the fractionation device are much lower, compared to SEC. The surface area of a SEC column is approximately 10^3^–10^4^ than that of the AF4 separation channel and the shear forces are in the order of 10^3^ higher in SEC [14]. In SEC, the risk of interaction with the column close packed material, and adsorption with the risk of column blockage, is often reduced by pH adjustments, or by applying higher salt concentrations or modifiers to the mobile phase. Proteins are in general prone to interaction with surfaces and can often contain supramolecular assemblies which can be shear sensitive or so large that they simply get stuck in a SEC column. AF4 can be used with a wide range of carrier liquids which can be chosen upon suitability for the sample to be measured in and with native conditions thereof [15–18]. These are some features that make AF4 attractive for the separation and characterization of biomacromolecules and biocolloids.

In AF4, as well as in SEC, separation leads to dilution of the analytes. The typical dilution factor is similar in AF4 and SEC and can be estimated for a typical separation of a monodisperse protein to be in the order of 50 × (depending on separation device properties, injected amounts, and flow rates). A concern for the coupling of AF4 to SAXS is that the concentration in fractions eluting from the AF4 channel would be too low to be adequately detected. AF4, similarly to SEC, has an upper limit on how much sample can be loaded onto the separation channel. If too much analyte mass is injected, then this leads to overloading and no, or impaired separation of analytes. The underlying reason is that the concentration of analytes becomes too high in the sample zone. In turn, this gives rise to interaction between analytes, and they are, thus, not able to establish a “correct” concentration profile away from the accumulation wall, based on their diffusion coefficient. Obviously, overloading should be avoided and the approach is to inject as much analyte as possible, i.e., maximize signal-to-noise ratio (S/N), while still avoiding overloading. The overloading may, thus, limit the possibility to increase the injected amounts should insufficient S/N be obtained in AF4-SAXS.

Biomolecules are generally weak scatterers of X-rays due to low electron density, resulting in low contrast, and at the same time sample concentration is generally kept at ideal solution conditions, to exclude the presence of structure factors. BioSAXS facilities are therefore constructed to provide a high X-ray flux, which is mandatory for dilute weak scatterers. The high X-ray flux may on the other hand produce radiation damage to the sample, which could result in aggregation and/or degradation [2, 19, 20]. When it comes to AF4-SAXS applications within the life sciences, these are better served when performed at a synchrotron facility in contrast to bench top SAXS instruments. For strong scatterers, like metallic nanoparticles and synthetic polymers, bench top SAXS instruments have been proven sufficient for adequate SAXS data collection in coupling to AF4 [21–23]. SEC-SAXS measurements on bench top SAXS instruments have been performed on proteins, but to acquire adequate S/N in the SAXS data so-called stop flow measurements were deployed, where part of the sample is measured under static, non-flowing conditions [24]. Stop flow measurements impair separation of the sample and the sample in its whole cannot be measured under continuous flow conditions.

In this study, we show that AF4 can be used for fractionation of proteins prior to on-line SAXS measurements and that a synchrotron SAXS flow-through capillary system can be used as an on-line detector for immediate scattering of the fractionated sample. For this purpose, we utilize the BioCUBE at the CoSAXS beamline at MAX IV Laboratory, Lund University, Sweden. We illustrate the setup by showing statistically adequate SAXS scattering data of bovine serum albumin (BSA), which is a well-documented and frequently used standard protein in laboratories today. We perform the same for a monoclonal antibody (mAb), as antibodies are of high interest today within biotherapeutics and especially within cancer immunotherapy [25].

## Methods

### Sample and carrier liquid preparation

The molecular weight of BSA is 66.5 kDa [26], the hydrodynamic radius (*R*_*h*_) is approx. 3.9 nm [27], and the radius of gyration (*R*_*g*_) is approximately 2.8 nm [28], depending somewhat on the buffer conditions used. BSA dissolved in aqueous buffer at pH 7.0 results in the formation of dimers, trimers, and possibly higher order oligomers.

The molecular weight of the mAb is approximately 148 kDa. Depending on the degree and type of glycosylation, the molar mass may range from 145 to 155 kDa.

BSA and mAb were prepared in the carrier liquid, Tris–HCL at pH 7.0, dissolved to a concentration of 3 mg/ml. Part of the mAb sample was enzymatically treated using IgGZero and the enzyme with a His-tag was removed by ion exchange chromatography after deglycosylation.

The AF4 carrier liquid of 25 mM Tris-(Hydroxymethyl)-aminomethane hydrochloride (Tris–HCl), pH 7.0, was prepared using MilliQ water (Merck Millipore, Darmstadt, Germany) and 0.02% w/w NaN_3_ added to prevent bacterial growth.

The analytical grade chemicals for preparation of samples and carrier liquid as well as bovine serum albumin (BSA) were purchased from Merck KGaA, Darmstadt, Germany. The mAb (trastuzumab) was supplied by Bernt Nilsson, Lund University, Sweden [29]. IgGZero was supplied by Genovis, Lund, Sweden [30].

#### AF4

The fractionation of proteins was performed on an Eclipse 3 + AF4 system (Wyatt Technology, Dernbach, Germany). The maximum crossflow setpoint allowed with the configuration of this system was 3.3 ml/min. The carrier liquid flow to the channel was delivered by an isocratic pump, the samples were injected by an autosampler, and the channel was connected to a UV detector at 280 nm, all of which were from the 1100-series from Agilent, Santa Clara, USA. The UV detector outlet was connected directly to the sample flow-through quartz capillary of the synchrotron source using a 0.8 m long, 250 µm inner diameter, PEEK capillary. Retrospective measurements of deglycosylated mAb were conducted on the same AF4 setup, but with UV detection and an on-line multiangle light scattering detector (MALS, Heleos II, Wyatt Technology). The software ASTRA (version 6.1.7.17, Wyatt Technology) was used for the acquisition of UV data and evaluation of UV-MALS data.

The carrier liquid was degassed by an in-line degasser and filtered before entering the channel and sample route by an in-line filter of mixed cellulose esters (MCE) type and of pore size 0.22 µm from Merck Millipore, Germany.

Two channels were used for the fractionation of samples, both from Wyatt Technology. BSA was fractionated on a mini channel (MC), with a 490 µm thick, wide spacer (490W) with a tip-to-tip length of 134 mm. The mAb was fractionated on a short channel (SC) with a 250 µm thick, wide spacer (250W) with a tip-to-tip length of 174 mm. Both channels were equipped with a membrane supporting frit from stainless steel. The membrane used in both channels was a regenerated cellulose (RC) membrane from Nadir GmbH, Germany, with a molecular weight cutoff of 10 kDa.

Because of the higher dilution of the sample in a high channel, BSA was injected at three different injection masses, 60, 150, and 300 µg, to check the performance of different eluting concentrations. Sixty micrograms of sample is slightly more than a typical amount of low molecular weight protein to inject. The detector flow was set to 0.5 ml/min, focus flow 2 ml/min, and injection flow 0.2 ml/min. Injection was performed in focus mode for 3 min, focused/relaxed for 3 min, and then eluted under constant cross flow of 2 ml/min.

The mAb was fractionated in an SC channel, injected at 100 µl, corresponding to 300 µg of injected mass. The detector flow was set to 0.5 ml/min, focus flow 3.3 ml/min, and injection flow 0.2 ml/min. Injection was performed in focus mode for 3 min, focused/relaxed for 3 min, and then eluted under constant cross flow of 3.3 ml/min.

The mAb was also measured in an AF4-UV-MALS setup using the same fractionation settings as described above and at an injected mass of 300 µg. A UV extinction coefficient of 1.36 ml/mg*cm has been used to determine concentrations for calculation of molecular weights. The MALS data was fitted using the Zimm model [31].

The resolution, *R*, was calculated by dividing the retention time difference between the monomer and dimer peaks with the corresponding average peak width.

### CoSAXS beamline, MAX IV Laboratory

The CoSAXS beamline at MAX IV has been constructed to deliver a high X-ray flux, 10^13^ at 12.4 keV, and a wavelength of *λ* = 0.99 Å. The data was collected on an Eiger2 4 M (Dectris) detector within an evacuated flight tube, positioned at two distances from the sample position: 4 m for BSA (3.2 × 10^−3^ < *q* < 0.32 Å^−1^) and at 8 m for mAb (1.31 × 10^−3^ < *q* < 0.15 Å^−1^), where *q* = 4πsin(*θ*)/*λ*, with 2*θ* the scattering angle and *λ* the X-ray wavelength. The flow cell consists of a 1.5 mm inner-diameter quartz capillary, with a 10 µm wall thickness.

### Analysis of SAXS data

ATSAS [3] program suite was used for the analysis of SAXS data and specifically CHROMIXS [10] was used to display frames obtained from the AF4-SAXS experiments and interactive buffer and sample region locating features were used. CRYSOL [32] was used for curve fitting using crystal structures. PRIMUS [4] was used for evaluation of *R*_*g*_ (according to Guinier), Porod volume, molecular weights as well as pair-distribution functions, P(*r*) [33], and Kratky plots [34]. DAMMIF [35] was used to calculate the total excluded volume of the BSA.

*R*_*g*_ was determined from the Guinier region in the range where 0.65 > *qR*_*g*_ < 1.3 in all cases, widely used for globular proteins and reported by Guinier and Fournet in 1955 [36]. The molecular weight from the Porod volume was estimated by division by 1.6 for BSA, which is considered a globular protein [37]. The molecular weight estimation from DAMMIF total excluded volume was concluded by division by 2, the globular protein assumption [37].

## Results

### AF4-UV-SAXS of BSA

**Fig. 1:**
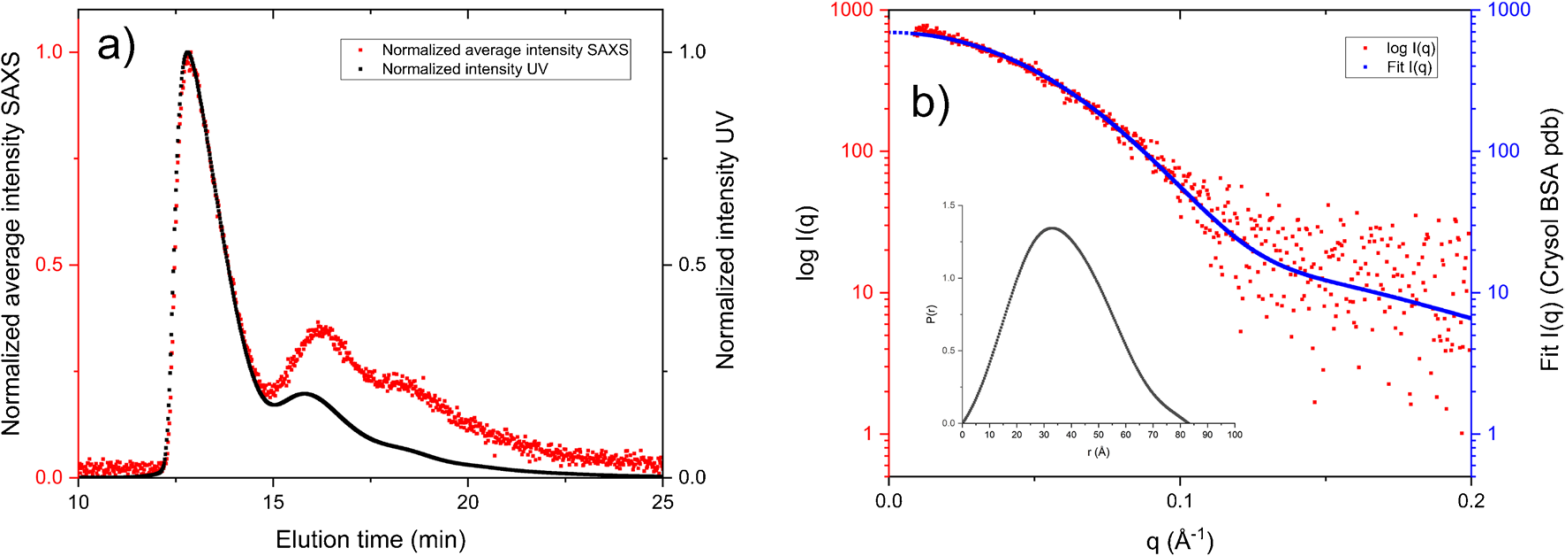
**a** Fractogram of BSA, 300 µg injected, with peak-maximum normalized average intensities of SAXS (red) and peak-maximum normalized intensity UV absorption at 280 nm (black) vs time. **b** SAXS data (red) from the monomer peak of BSA and the corresponding fit to the crystal structure of monomeric BSA (PDBID: 3V03), using CRYSOL (blue) in a log I(*q*) vs *q* plot, P(*r*) vs *r* inset

The results from AF4-UV-SAXS of BSA are presented in Fig. 1. Three populations are visible in the fractogram in Fig. 1a corresponding to (in elution order) monomer, dimer, and trimer/larger species. It should be noted that by comparing the signals for the monomer peak (approx. 13 min) the sample zone broadening between the UV-detector and SAXS capillary is negligible. The results from fitting of the SAXS data are shown in Table 1. The BSA monomer data was evaluated for all injected masses and the dimer data for the two highest masses injected, i.e., 150 and 300 µg. The dimer concentration for the lowest mass injected, 60 µg, was too low to produce satisfactory SAXS data and could not be resolved due to poor S/N. Figure 1 shows the fitted data on top of the experimental SAXS data from injection of 300 µg together with the pair-distribution function P(*r*) vs *r*. The P(*r*) function shows a Gaussian distribution as expected for a globular protein.

**Table 1:** Results from the evaluation of SAXS data from elutions of BSA, injected at 60, 150, and 300 µg respectively. R_g_ determination was robust with different methods and in agreement with the expected values. The experimentally determined molecular weights yield values within less than 10% of the theoretical value of 66.5 kDa from the 300 µg injected mass for both the dimer and monomer

BSA, injected mass	Guinier, Rg [Å], (Std)	P(*r*), Rg [Å]	DAMMIF Rg [Å]	Porod volume [Å^3^]	Mw (Porod) [kDa]	DAMMIF Total excluded volume [Å^3^]	Mw (DAMMIF) [kDa]	Total quality estimate from ATSAS
Monomer, 300 µg	27.5 (± 0.3)	27.5	27.8	99.7	62.3	123	61.7	0.93
Monomer, 150 µg	28.6 (± 0.4)	28.6	28.0	92.6	57.9	118	59.0	0.92
Monomer, 60 µg	28.4 (± 0.4)	28.5	28.5	88.4	55.2	117	58.4	0.91
Dimer, 300 µg	39.3 (± 4.0)	39.3	39.4	195	122	270	135	0.84
Dimer, 150 µg	44.5 (± 1.8)	44.6	44.6	221	138	277	138	0.84

**Fig. 2:**
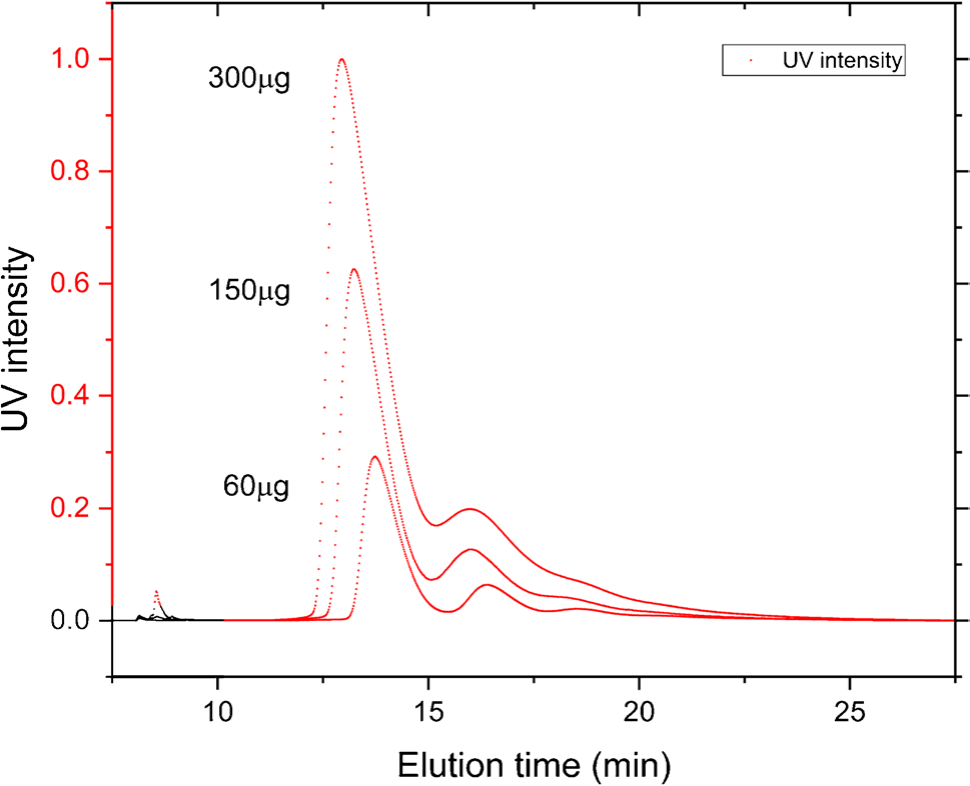
UV fractograms of BSA injected at 60, 150, and 300 µg. The injected masses of 150 and 300 µg show decrease in resolution and slightly shorter elution times, approximately 45 s

At 150 and 300 µg injected mass, slight overloading was observed with a decrease of elution time of 45 s for the monomer peak compared to the 60-µg injection (Fig. 2). The resolution, *R*, for 60, 150, and 300 µg injected were 2.4, 1.5, and 1.3 respectively (Fig. 2).

### AF4-UV-SAXS of monoclonal antibody

**Fig. 3:**
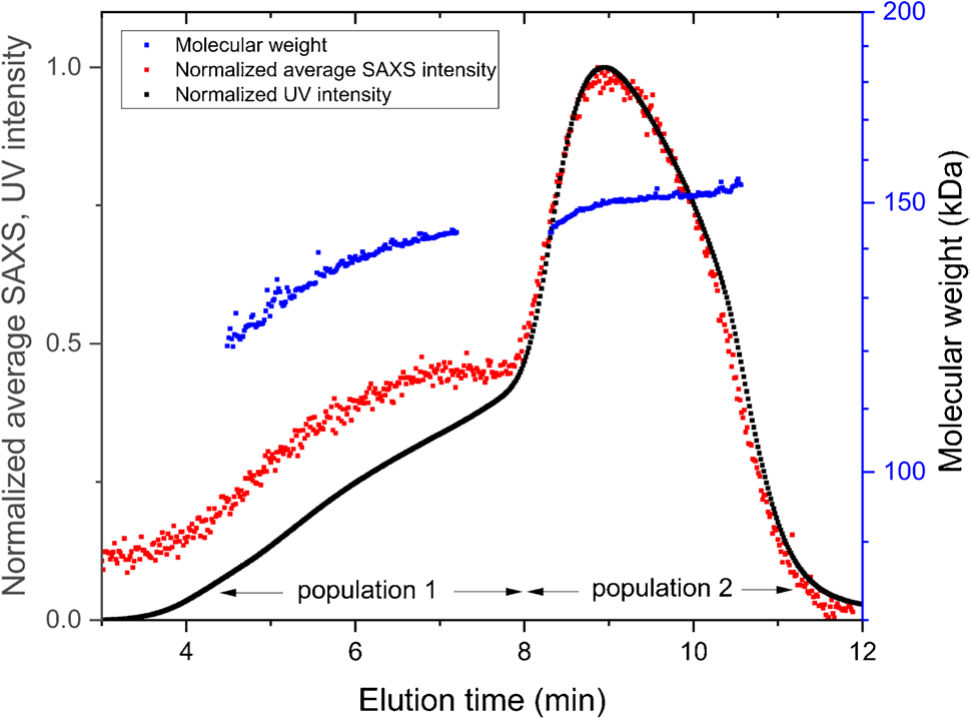
Fractogram of mAb, 300 µg injected, with peak-maximum normalized average intensities of SAXS (red) and UV absorption at 280 nm (black) vs elution time. Overlayed molecular weight in log scale (blue), increasing from 120 kDa to approximately140kDa in population 1 and approximately constant at 150 kDa over population 2

Figure 3 shows AF4-UV-SAXS fractograms and molecular weight for the mAb at a sample mass of 300 µg. The molecular weights were derived from measurements using AF4-UV-MALS. There are two distinct regions in the fractogram, from 4 to 8 min and 8 to 12 min, referred to as populations 1 and 2. Population 1 shows molecular weights from approximately 120 to 140 kDa. Population 2 shows a molecular weight of 150 kDa.

The increasing molecular weight over population 1 needed further investigation since the molecular weight of monomeric mAb is approximately 148 kDa. A deglycosylation of the mAb was performed to investigate whether the glycosylation pattern in the total population was varying.

**Fig. 4:**
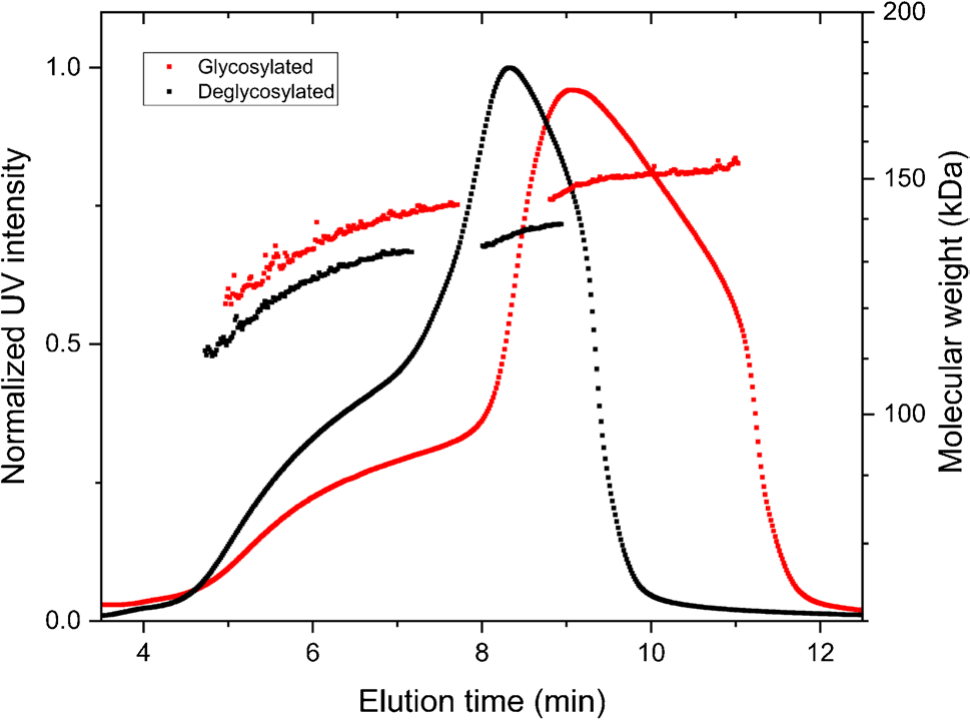
Fractionations of glycosylated (red) and deglycosylated (black) mAb. Molecular weights follow the same color coding as the UV signal. Molecular weight in log scale, increasing from 110 kDa to approximately130kDa for population 1 and approximately 135–140 kDa over population 2 for the deglycosylated sample. For the glycosylated sample molecular weights are 120–140 kDa over population 1 and 150 kDa for population 2

Figure 4 shows UV fractograms of glycosylated and deglycosylated mAb, the shift in molecular weights and retention times after deglycosylation. The results show an overall decrease of molecular weights, in both populations, of approximately 10 kDa, and an unchanged fractogram profile, i.e., two populations, but shifted to lower elution times.

**Fig. 5:**
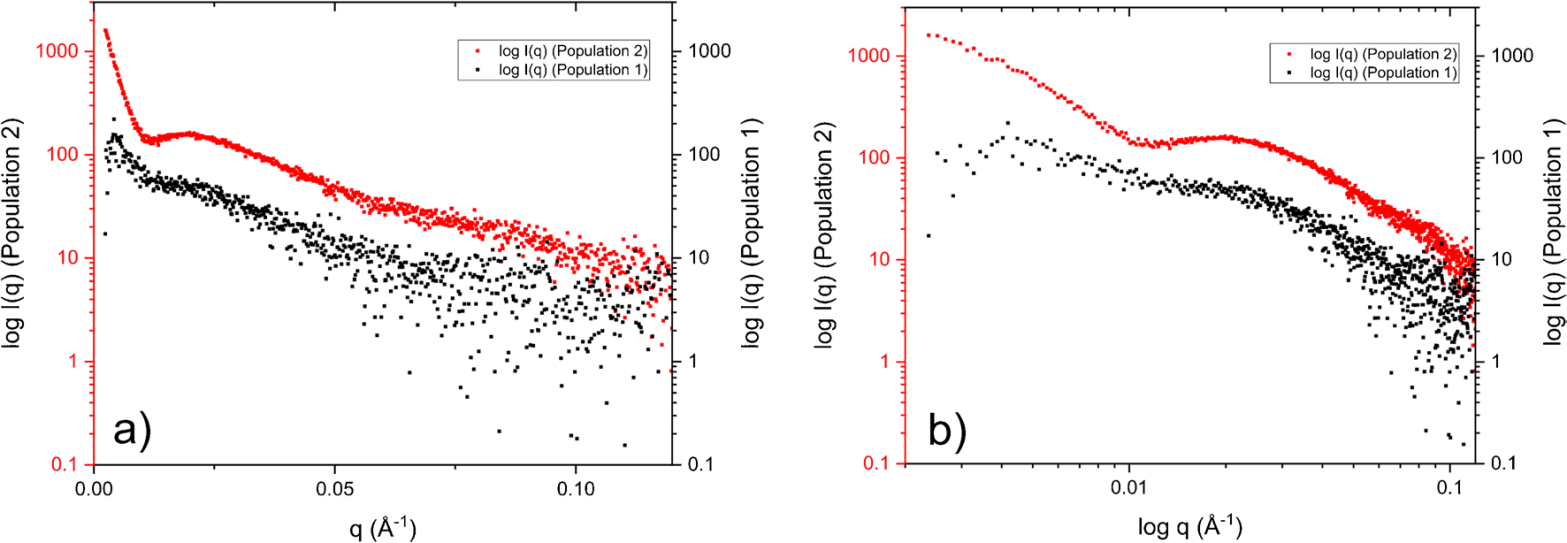
I(*q*) data, from population 1 (black, elution time 4–8 min) and population 2 (red, elution time 8–12 min) of the AF4 fractogram, in log I(*q*) vs *q* (a) and log I(*q*) vs log *q* (b) plots. Obvious artifacts visible in the low *q* region for both populations

The normalized and background subtracted SAXS data was analyzed by comparing results from the two populations. Figure 5 a and b show the SAXS data in log I(*q*) vs *q* and log I(*q*) vs log *q*. An increasing I(*q*) was observed in both populations from *q* < 0.01 Å^−1^. In the data from population 2 (red curves in Fig. 5), there is also a distinct local minimum before the increase at lower *q*.

**Fig. 6:**
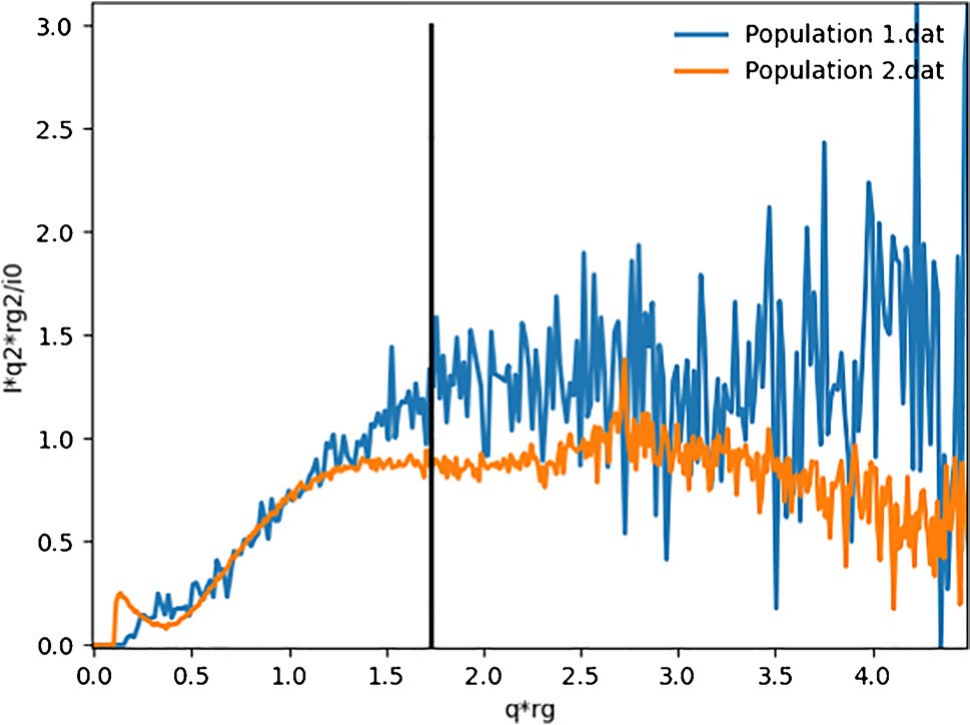
*q*^2^-Normalized Kratky plots for populations 1 (blue) and 2 (orange) respectively. The black line indicates q*Rg = √3, where globular proteins have their maximum

The Kratky plots in Fig. 6 indicate flexible structures in both populations. For population 2, a weakly pronounced minimum is observed after the first maximum, possibly indicating a folded conformation. The Kratky plot for population 1 is very noisy but seems to decay to 0 at higher *q*, which would indicate a flexible structure rather than an unfolded/intrinsically disordered structure [37].

**Fig. 7:**
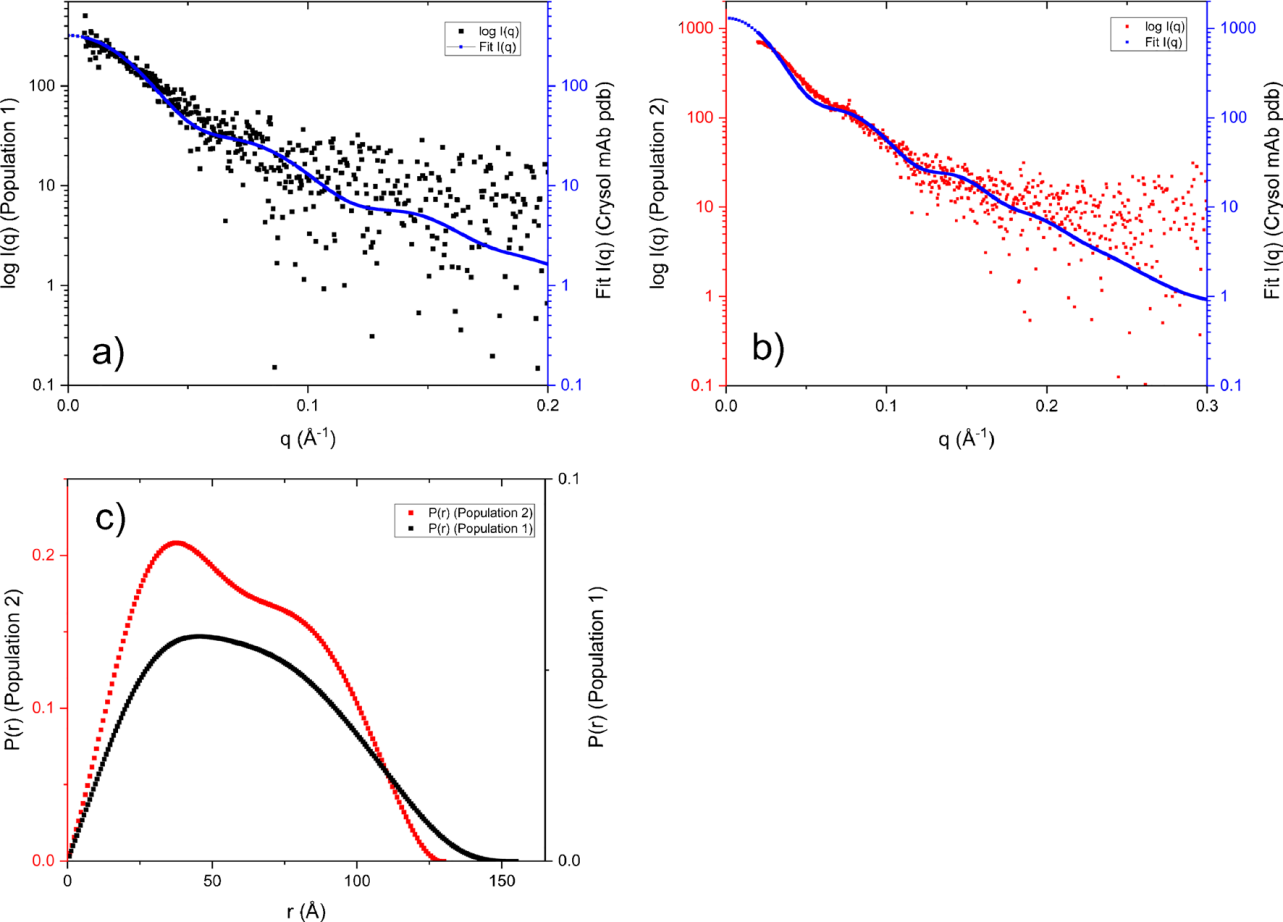
**a** I(*q*) and fitted data to the crystal structure of mAb from population 1 (4–8 min) of the fractogram in Fig. 3. **b** I(*q*) and fitted data to the crystal structure of mAb from population 2 (8–12 min) of the fractogram in Fig. 3. **c** P(*r*) functions of populations 1 and 2 respectively

The SAXS data analysis from both populations was conducted after exclusion of data from the low-*q* range in the analysis, from *q* = 0.015 Å^−1^ and below for population 1, and from *q* = 0.02 Å^−1^ for population 2. These analyses are presented in Fig. 7 a and b, showing log I(*q*) versus *q* and the fitted functions from CRYSOL using experimental data and the X-ray structure of mAb (PDB ID:1IGT) overlayed. In the same figure, the corresponding pair-distance distribution functions are represented in a P(*r*) vs *r* graph (Fig. 7c). Guinier regions were found for both populations in this higher *q*-range and with 0.42 < *qRg* < 1.3 for population 1 and 0.64 < *qRg* < 1.3 for population 2. Table 2 shows the parameters from the fitting of the SAXS data analysis. There is a deviation between MALS and Porod volume-determined molecular weights. This deviation is within 10% and can be related to the difficulties in determining molecular weights for flexible proteins from the Porod volume.

**Table 2:** Results from AF4-SAXS on mAb, with a separation channel massload of 300 µg

Population	Guinier R_g_ [Å]	P(*r*) R_g_ [Å]	Dmax (Å)	Porod volume [nm^3^]	Mw (Porod) [kDa]	Total quality estimate from ATSAS
1	48.0	48.1	156	255	159	0.94
2	44.9	44.9	144	215	135	0.94

## Discussion

BSA was used in this work for general recognition and the results are satisfactory in terms of size and molecular weight. This is an important piece of the puzzle to show the potential of AF4-SAXS on proteins, and the results show that the eluting concentration from the AF4 channel is sufficient to produce SAXS data with adequate S/N. It should also be mentioned that during these trials the SAXS flow-through capillary was not cleaned in direct conjunction to the experiments performed. Also, the fractionation of BSA was performed in a channel where a 490 µm spacer constituted the channel height. With a new capillary and lower channel height, an increase in S/N is expected as dilution would be lower, which means that the dimer of 60 µg injections could possibly have been evaluated as well. An injected mass of 60 µg represents a typical amount to inject for AF4 fractionations of BSA.

The mAb sample used in this work shows more than just monomeric content, which is why it was chosen for this work. The molecular weight of mAb is approximately 150 kDa, consistent with the result in Fig. 3, for population 2. It is important to notice that there are no oligomers or aggregates visible in the fractogram; there is no indication of coelution from the AF4 channel. Coelution can be a consequence of overloading when analytes are hindered to reach their equilibrium height position in the AF4 fractionation channel prior to elution. Coelution can also appear when steric/hyperlayer elution occurs in parallel to Brownian mode, the former due to the physical sizes of analytes [38]. Steric/hyperlayer elution occurs for analytes from *R*_*h*_ ≈ 500 nm and above. The consequence of coelution is simultaneous elution of a range of sizes, which will impair the light scattering signal, resulting in overestimation of or erratic molecular weights from MALS [39]. However, in this case, there is a clear connection between increasing hydrodynamic radius (increasing retention time) and increasing molecular weight, which reaches a plateau for population 2 and is consistent with the molecular weight of mAb.

There can be several explanations for the molecular weight increase with elution time over population 1. Two possible explanations are protein degradation and/or differences in glycosylation. A variation in glycosylation patterns in antibodies is well known, while a light chain of a mAb corresponds to approximately 25 kDa, which is a difference within the observed molecular weight span in population 1 (Fig. 3). To exclude the possibility of a difference in glycosylation pattern in the mAb assembly, fractionation of a deglycosylated assembly was performed. In Fig. 4, a comparison of the original mAb and the deglycosylated version is shown. This indicates that population 1 does arise not only from differences in glycosylation but likely also from degradation of peptide linkages in the mAb. Conclusions can hardly be drawn, though, since both light chain degradation and glycosylation variations contribute to the molecular weight. As AF4 fractionates according to hydrodynamic size and *R*_*h*_ is directly proportional to the elution time when utilizing constant crossflow [12], the observed difference in elution time (4–8 min for population 1 and 8–12 min for population 2) corresponds to a difference in *R*_*h*_ of a factor 1.3–2.5. A change in *R*_*h*_ of factor two corresponds, e.g., to the difference between monomer and dimer in a protein assembly.

The results for population 1 show higher values than those for population 2 for *R*_*g*_ and *D*_*max*_. This together with the differences observed in the P(*r*) functions in Fig. 6 indicates an elongation of the molecule upon degradation. As discussed above, the fractograms show mAb in population 1 degraded in such a way that *R*_*h*_ decreases, resulting in shorter elution times. When parts of the three domains of the mAb degrade, this could enable access to a formerly excluded volume for the remaining domains (linked by flexible linkers), with the consequence of an elongation. This is also supported by an increase in *R*_*g*_ and *D*_*max*_ and a decrease in *R*_*h*_. These results can be concluded by a comparison to the expected scattering of the mAb, the approximate monomer size is known and well documented [40]. The results, from the fits with the low *q*-range excluded, turn out to appear valid in this case.

There are several possible reasons for the behavior in the low-*q* region: aggregation, intermolecular interactions, scattering artifacts in proximity to the beam stop, radiation damage and flexible linkers in the mAb [37]. With the data collected, it is not possible to come to a definite conclusion regarding the cause. In other publications of SAXS data from mAb, the lower limit of the published *q*-range is about 0.02 Å^−1^ [41, 42] and does not show as low *q* as in the current study.

Intermolecular interactions are unlikely since the measurements were performed at dilute concentrations, and as mentioned above, there are no oligomers or aggregates visible in the fractograms from AF4-UV or AF4-UV-MALS. Nor are scattering artifacts related to the beam stop a realistic explanation as such artifacts were not visible prior to or after the mAb fractionation, which excludes this as an explanation to the scattering at *q* < 0.02 Å^−1^.

Flexible proteins, e.g., IgG, have been reported to produce deviant Guinier curvatures [37]. This could be explained by the flexible linkers between the domains of antibodies, which to some extent have freedom to shift their relative position independently [43]. This can explain why the Guinier region can be more difficult to determine for a flexible protein. Depending on the length scale studied, either will be influenced by contributions from the individual domain size and the entire protein size, respectively. At the same time, the entire protein size can be somewhat unprecise due to its flexibility. Altogether, this can give rise to Guinier region fluctuations or averaging. However, and which is further explained below; in this work, we did not experience any problems identifying linear Guinier regions for the mAb. The Kratky plot in Fig. 6 clearly indicates flexible structures in both populations 1 and 2 though.

Radiation damage to protein in solution subjected to synchrotron radiation has been observed before and is a known problem. With the BSA sample characterized in this work, no indications of radiation damage were observed, although measured at the same X-ray flux and energy. Possibly, the mAb is more sensitive to the radiation dose compared to BSA. This means that we cannot exclude reduction of the disulfide bonds in the mAb by the X-ray beam. In turn, these effects could cause artifacts such as aggregation.

The *q*-range used here was not low enough to capture the full Guinier range for these seemingly larger structures. Nevertheless, an attempt to fit a larger structure was performed using SASview (http://www.sasview.org/) and a simple sphere model (not shown) with the result of *R*_*g*_ = 35–45 nm over the whole AF4 fractogram. Despite the likely aggregation of mAb in the flow-through capillary, only minor influence in the scattering pattern of native mAb monomer was found when compared with the X-ray crystal structure (PDB ID: 1IGT).

The SAXS data deviation from a plateau in the expected *q*-range (< 0.02 Å^−1^) for monomeric mAb becomes more and more pronounced as elution time increases, and the deviation is at its highest for the data collected in population 2, where the concentration is also at its peak. The increasing concentration of sample eluting, with elution time, shown by the fractogram in Fig. 3, would increase the risk of radiation damage [44]. For future attempts to analyze mAbs using AF4-SAXS, it is recommended to perform more thorough investigation of sample concentration and X-ray flux limitations for radiation damage in the eluted fractions.

We have shown that statistically adequate data for proteins can be obtained using AF4 coupled on-line to synchrotron SAXS. Serial coupling of AF4 to UV and the SAXS capillary resulted in negligible band broadening of eluting fractions. The results show that characterization of the proteins was achieved over the size distribution obtained from fractionation with AF4. BSA was injected at different masses and even the lowest injected mass of 60 µg was shown adequate to determine size and molecular weight of the monomer. A monoclonal antibody was used as a second model system. Adequate signals from the eluate are obtained also in this case, enabling the determination of size and molecular weight.

The AF4-UV-SAXS results for the monoclonal antibody show the presence of what appears to be aggregated structures. Most likely, these structures originate from radiation damage in the SAXS capillary, as they were not present in the UV fractogram prior to SAXS, nor when MALS detection was used. Aggregates of sizes observed here would have eluted as a separate population from the AF4 fractionation channel. This shows one strength of the technique presented here, as we can conclude that the aggregates were not part of the original sample, which would not necessarily have been achievable in a batch mode measurement.

It is possible that higher flow rates from the fractionation channel could help avoid the negative effects of radiation damage, as the residence time in the capillary will be reduced. It is possible that a decrease in X-ray flux would effectively do the same, while still achieving adequate data from mAb after AF4 fractionation. Collecting data frames less frequently could also be a possible solution, allowing more time for fresh solution to enter the X-ray path.

In conclusion, with this proof of concept of AF4-SAXS, we see high potential in applying this technique to, for instance, areas like pharmaceutics and biologics specifically, especially for complex biomolecule assemblies where a low fractionating shear force and low internal surface area in the separation device are required not to induce structural changes and/or to samples containing large analytes or wide size ranges.
